# Feasibility and Acceptability of a Finger‐worn Device for Daily Stress Assessment in Culturally‐matched Home Care Workers of Individuals with ADRD: Preliminary Findings

**DOI:** 10.1002/alz70858_104382

**Published:** 2025-12-26

**Authors:** Hyejin Kim, Olimpia Paun, Eunbee Liu, Jaehyuk Song, Sruthi Ramadurai, Myunghee Kim

**Affiliations:** ^1^ Rush University College of Nursing, Chicago, IL, USA; ^2^ Rush University Medical College, Chicago, IL, USA; ^3^ University of Illinois Chicago College of Engineering, Chicago, IL, USA

## Abstract

**Background:**

Culturally matched home care workers (HCWs) for immigrants with ADRD face high stress from addressing cultural care needs. While wearables measuring heart rate variability (HRV) show promise as stress indicators, their feasibility and acceptability for daily use in HCWs remain unclear. Focusing on Korean‐American HCWs as an exemplar of an ethnic minority, we presented preliminary findings on using a finger‐worn device for longitudinal stress monitoring.

**Methods:**

We included Korean‐American HCWs (≥6 months of caregiving experience) of Korean immigrants with ADRD who owned a smartphone. HCWs caring for their own family members were excluded. Data collection began in September 2024. Participants wore Ōura rings for 21 consecutive days (7 days for baseline data establishment and 14 days for analysis). We measured recruitment and enrollment rates, completion rates of data collection, HRV data quality (valid data), and participants’ perceived ease of use and comfort (rated on a scale from 1 [very unlikely] to 5 [very likely]). HRV data were accessed through the Ōura application programming interface and analyzed using Python.

**Result:**

Of the 27 HCWs referred to the study, 7 (25.93%) were ineligible and 20 (74.07%) were invited. Of those invited, 18 (90.0%) consented to the study. Three (16.67%) dropped out afterward, leaving 15 (83.33%) HCWs who started the study. Fourteen participants completed 21 days of wearing the rings, while one participant remains ongoing. Among the 14 HCWs who completed the study, 8 (57.14%) had valid HRV data for all 14 days, whereas 6 (42.86%) had 1 to 4 invalid data despite completing the study. The average scores for ease of use and comfort of the ring were 4.21 (SD=1.05; range=2‐5) and 3.86 (SD=1.03; range=2‐5), respectively.

**Conclusion:**

The study demonstrates the feasibility and acceptability of Ōura for daily HRV monitoring among Korean‐American HCWs caring for Korean immigrants with ADRD. Future studies with larger sample sizes are necessary to validate these findings. Additionally, an in‐depth understanding of the sources of stress and unmet needs in this HCW population is critical to inform culturally tailored interventions.

